# Effect of Obesity and Decompressive Laparotomy on Mortality in Acute Pancreatitis Requiring Intensive Care Unit Admission

**DOI:** 10.1007/s00268-012-1821-8

**Published:** 2012-10-10

**Authors:** Philip J. B. Davis, Karim M. Eltawil, Bassam Abu-Wasel, Mark J. Walsh, Trevor Topp, Michele Molinari

**Affiliations:** Division of General Surgery, Department of Surgery, Queen Elizabeth II Health Sciences Centre, Dalhousie University, Room 6-302 Victoria Building, 1276 South Park Street, Halifax, NS B3H 2Y9 Canada

## Abstract

**Background:**

Controversy still exists on the effect that obesity has on the morbidity and mortality in severe acute pancreatitis (SAP). The primary purpose of this study was to compare the mortality rate of obese versus nonobese patients admitted to the ICU for SAP. Secondary goals were to assess the potential risk factors for abdominal compartment syndrome (ACS) and to investigate the performance of validated scoring systems to predict ACS and in-hospital mortality.

**Methods:**

A retrospective cohort of adults admitted to the ICU for SAP was stratified by their body mass index (BMI) as obese and nonobese. The rates of morbidity, mortality, and ACS were compared by univariate and multivariate regression analyses. Areas under the curve (AUC) were used to evaluate the discriminating performance of severity scores and other selected variables to predict mortality and the risk of ACS.

**Result:**

Forty-five patients satisfied the inclusion criteria and 24 (53 %) were obese with similar characteristics to nonobese patients. Among all the subjects, 11 (24 %) died and 16 (35 %) developed ACS. In-hospital mortality was significantly lower for obese patients (12.5 vs. 38 %; *P* = 0.046) even though they seemed to develop ACS more frequently (41 vs. 28 %; *P* = 0.533). At multivariable analysis, age was the most significant factor associated with in-hospital mortality (odds ratio (OR) = 1.273; 95 % confidence interval (CI) 1.052–1.541; *P* = 0.013) and APACHE II and Glasgow-Imrie for the development of ACS (OR = 1.143; 95 % CI 1.012–1.292; *P* = 0.032 and OR = 1.221; 95 % CI 1.000–1.493; *P* = 0.05) respectively. Good discrimination for in-hospital mortality was observed for patients’ age (AUC = 0.846) and number of comorbidities (AUC = 0.801). ACS was not adequately predicted by any of the clinical severity scores (AUC = 0.548–0.661).

**Conclusions:**

Patients’ age was the most significant factor associated with mortality in patients affected by SAP. Higher APACHE II and Glasgow-Imrie scores were associated with the development of ACS, but their discrimination performance was unsatisfactory.

## Introduction

The clinical presentation of acute pancreatitis (AP) ranges from mild edematous to severe acute necrotizing pancreatitis (SAP) [1]. Edematous pancreatitis usually resolves without major consequences, whereas SAP is associated with considerable morbidity and mortality [1]. The death rate has significantly decreased over time, from 60 to 80 %, during the early 20th century, to 20–30 % in recent years [1–4]. Still, prediction of the clinical course of these patients remains challenging. Several models are used to stratify the severity of the disease at presentation [5–9], but they lack sufficient predictive granularity [10, 11]. One of the reasons for this limitation is the fact that they may not capture all of the relevant preexisting medical and physiological conditions that might influence patients’ prognosis [11].

With the increasing prevalence of obesity [12–18], there is growing evidence that obese patients with SAP might have worse outcomes in comparison to patients with normal body mass index (BMI) [19–24]. The higher mortality of obese patients is thought to be due to the additive effect of more extensive fat necrosis [25], chronic up-regulation of the inflammatory response [26], and subsequent increased risk of multiorgan failure (MOF) and abdominal compartment syndrome (ACS) [22]. However, further studies are necessary, because some authors have failed to confirm these findings and found no differences between obese and nonobese patients [22, 25–27]. Studies on clinical outcomes of patients admitted to the ICU for SAP are scarce, and only a few investigated the role of obesity in the development of ACS. In addition, there is a lack of studies to evaluate the performance of scoring systems to predict the development of ACS in the presence of SAP.

In view of these limitations, the primary purpose of this study was to test the null hypothesis for in-hospital mortality and the development of ACS between obese and nonobese patients affected by SAP and admitted to the ICU. Secondary goals were: to evaluate the incidence of ACS, to examine risk factors for ACS, to assess the outcomes of patients undergoing decompressive laparotomy (DL), and to investigate the performance of validated scoring systems (e.g., Ranson, APACHE II, Glasgow-Imrie Scale, SOFA) and selected patients’ characteristics (e.g., age, BMI, gender, number of comorbidities) to predict ACS and in-hospital mortality.

## Patients and methods

### Study design

A cohort of patients affected by SAP was retrospectively identified at the Queen Elizabeth II Health Sciences Centre, Halifax, Nova Scotia (Canada). All patients admitted to the ICU between July 1, 2005 and June 30, 2009 were screened for eligibility according to the study protocol approved by the local ethics review board (ERB). The International Classification of Diseases Version 9 (ICD-9) code 577.0 was used to identify patients with SAP from a prospectively maintained electronic database. The diagnosis of AP was confirmed by threefold elevation of serum amylase and lipase levels during the 72 h period preceding their admission to the ICU. For each patient, the following variables were collected: age, gender, body weight, height, number of comorbidities (ICD-9 codes), date of admission and discharge from ICU, development of ACS, intra-abdominal pressures measured in patients with suspected IAH, need for DL, time interval from diagnosis of ACS to DL, surgical technique used to manage the laparostomy site, postoperative abdominal wall complications, and overall mortality rate.

Severity of AP at the time of ICU admission was determined by validated prognostic models: Acute Physiology and Chronic Health Evaluation II score (APACHE-II; range 0–67) [28, 29], Glasgow Scale (GS; range 0–8), Sepsis-related Organ Failure Assessment score (SOFA; range 0–24) [8], Ranson score (range 0–11) [5, 30], and Charlson Comorbidity Index (CCI) (range 0–36) [31, 32]. Predicted mortality of the study population was calculated by using normograms or validated logistic equations of both Ranson criteria and APACHE-II scores [33, 34].

### Sample size calculation

Sample size calculation was based on the estimated ICU mortality of nonobese patients that in previous studies was reported to be up to 30 % [1–3]. The expected mortality of obese patients was estimated by doubling the death rate of normal weight individuals affected by SAP as published in a recent meta-analysis [21]. Using these premises, a total number of 42 subjects were needed to reach a power of 0.8 with a two-tail alpha level of 0.05.

### Inclusion and exclusion criteria

Patients included in this study satisfied the following criteria: adult age (older than 18 years), a primary diagnosis of SAP by the Atlanta criteria [35, 36] and requiring admission to ICU for at least one of the following conditions: hypotension, renal failure, respiratory insufficiency, cardiac dysfunction, disseminated intravascular coagulation, or gastrointestinal hemorrhage [37].

Exclusion criteria were: pregnant women, patients younger than 18 years, diagnosis of acute or chronic pancreatitis, recent traumas, or surgical interventions responsible for the development of AP.

## Definitions

### Severity of acute pancreatitis

SAP was defined as AP in the context of new onset of organ failure and/or local complications according to the revised Atlanta classification of AP [35, 36] _ENREF_37. Patients with SAP were defined as affected by at least one organ failure that lasted more than 48 h or who developed complications leading to death.

On the first day of admission to ICU, the severity of AP was measured by utilizing the Glasgow-Imrie score [6], the APACHE II [7], and the SOFA [8] scores. A fourth prognostic model, the Ranson score [5], was calculated by the combination of patients’ age, laboratory, and clinical variables obtained at the time of hospital admission and 48 h later. Predicted mortality rate of the study population was calculated by using the Ranson score and the adjusted APACHE II logistic regression model [34]:

$$ {\text{Predicted death rate }} = - 3, 5 1 7 { } + \, \left( {\text{APACHE II score}} \right)\; \times \;0. 1 4 6 { } + \, 0. 50 1. $$

Calculations were performed by accessing the on line calculator available at the Societe’ Francoise d’Anesthesie et de Ranimation webpage [38].

### Abdominal compartment syndrome

Abdominal compartment syndrome was defined as the presence of intra-abdominal pressure equal or higher than 20 mmHg in association with acute organ failure [39]. Intraperitoneal pressure was measured by inserting a standard Foley urinary catheter of at least 16 Fr into the patient’s bladder, which was then filled with 25–30 mL of saline solution. Measurements of the intra-abdominal pressure were obtained at the end of the expiration and with the patients in supine position [40] by connecting the Foley catheter to a digital pressure transducer after clamping its outflow channel. The pubic symphysis was considered the reference level [41].

Alternatively, ACS was diagnosed during clinical examinations when patients experienced severe abdominal distension and at least one of these other conditions: (1) tachycardia and/or hypotension despite elevation of the central venous pressure (CVP), (2) tachypnea and/or elevated peak inspiratory pressures when on ventilator support with refractory hypoxemia and/or hypercapnia, (3) renal dysfunction not responsive to intravenous diuretics or dopamine infusion [40].

### Obesity

Body mass index (BMI; Kg/m^2^) ≥30 was used as a cutoff point to identify obese patients according to the definition proposed by the World Health Organization [11].

### Decompressive laparotomy

Decompressive laparotomy was defined as any surgical intervention designed to reinstitute the physiological abdominal wall compliance. This was obtained by interrupting the skin, fascia, and peritoneum along a midline abdominal incision extending from the xiphoid process of the sternum to the suprapubic area in combination with the anterior wall muscles if necessary [42]. During DL, the intra-abdominal organs were inspected and released from the tension of the enclosed cavity until a satisfactory cardiopulmonary response was obtained. All patients underwent exposure of edematous intestinal loops, omentum, and solid organs, and none was treated by subcutaneous linea alba fasciotomy or transverse laparostomy [40, 43, 44]. Intraperitoneal drains were placed at the discretion of the surgeons and no pancreatic debridement was performed during DL.

### Temporary abdominal closure

Temporary abdominal closure was defined as any technique used to close the abdominal cavity provisionally by creating a laparostomy that allowed decompression of the intraperitoneal organs but preventing their contamination, hypothermia, and fluid losses [45]. Available techniques for the provisional abdominal wall closure were: (1) placement of absorbable mesh material consisting of polyglactin 910 (Vicrlyl-mesh, Ethicon^®^) or polyglicolic acid (Dexon-mesh, Dexon^®^) [46, 47], (2) coverage of the intra-abdominal organs with expanded polytetrafluoroethylene (ePTFE) foils [48, 49] or adhesive (Opsite plastic dressing, 3 M, Tegaderm^®^) and nonadhesive plastic layer derived from irrigation bags (e.g., Bogota-bag) [49, 50], and (3) combining adhesive plastic foils with a polygalactin sponge (V.A.C KCL^®^) and delivering a constant negative pressure of 100–150 mmHg applied via tubes connected to a computerized portable vacuum device [51–53].

### Length of hospital stay and mortality

Hospital stay was defined as the number of days that each patient spent at the tertiary medical centre. All causes of mortality during this period of time were considered direct consequences of SAP. Deaths that might have occurred after the patients were discharged home or to rehabilitation centers or to long-term facilities were not measured as not traceable by the investigators.

## Statistical analysis

Summary statistics were constructed for the baseline values, using frequencies and proportions for categorical data, and mean and standard deviation (SD) for continuous variables. Categorical outcomes were analyzed by using chi-square test or Fisher’s exact test when appropriate. Continuous variables were compared by using Mann–Whitney or Kruskal–Wallis test. Univariate logistic analysis also was preformed to look for possible associations with morbidity, mortality, and the development of ACS (age, obesity, number of comorbidities, Ranson score, SOFA score, Glasgow-Imrie score, APACHE II score). Multivariable logistic regression analyses were performed using step-wise techniques to explore predicting factors for the development of ACS and mortality. The stepwise procedure was set at the threshold of 0.1 for inclusion and 0.05 for exclusion. Discrimination was analyzed as the capacity of prognostic models or clinical parameters to distinguish high-risk from low-risk individuals for hospital mortality and development of ACS. Discrimination of predicting factors was then assessed by Receiver Operating Characteristic (ROC) curves. Excellent discrimination was defined if the area under the curve (AUC) of ROC was ≥0.8, good discrimination was defined when the AUC was between 0.7 and 0.8 and poor discrimination when the AUC was <0.7. ROC curves were compared to the reference line associated with AUC = 0.5. Statistical analysis was performed by using SPSS^®^ software (Version 19, SPSS, Inc., Chicago, IL), and all tests were two-tailed and considered significant when *P* < 0.05.

## Results

### Study cohort

During a 4-year period, a total of 72 patients were admitted to ICU with the diagnosis of AP. After reviewing their medical files, 27 subjects were excluded, because they failed to satisfy the revised Atlanta criteria for SAP published in 2008 [35, 36] as their organ failure resolved within 48 h. The remaining 45 patients represented the study population and their demographic and clinical characteristics are summarized in Table 1. Mean time from hospital admission to ICU admission was 3.8 days.

**Table 1:** Patients’ characteristics and clinical variables at admission to ICU and 48 h later for calculation of Ranson score (total patients = 45)

Variable	Value
Demographic	
Age, year (mean, SD)	59 (13.3)
Male gender (no. patients, %)	36 (80)
Hospital mortality (no. patients, %)	11 (24.4)
Length of overall hospital stay, days (mean, SD)	85.9 (107.8)
Etiology of pancreatitis (no. patients, %)	
Gallstone	24 (53.3)
Alcohol	12 (26.7)
Idiopathic	7 (15.6)
Postendoscopic cholangiopancreatography (ERCP)	2 (4.4)
Clinical variables at admission to ICU	
BMI (mean, SD)	30.6 (5.2)
Obesity (BMI > 30) (no. patients, %)	24 (53)
Body temperature, Celsius (mean, SD)	37.8 (0.7)
Heart rate/min (mean, SD)	111.5 (19.3)
Systolic blood pressure, mmHg (mean, SD)	128.4 (20.1)
Diastolic blood pressure, mmHg (mean, SD)	68.2 (13.6)
Respiratory rate/min (mean, SD)	19.7 (8.9)
Laboratory variables at admission to ICU	
White blood cells, 10^3^/μL (mean, SD)	14.9 (4.5)
Platelets, 10^3^/μL (mean, SD)	221.4 (139)
Creatinine, μmol/L (mean, SD)	217.7 (160)
Glucose, mmol/L (mean, SD)	8.9 (4.4)
LDH, U/L (mean, SD)	479.3 (335.8)
AST, U/L (mean, SD)	90.4 (74.3)
Arterial pH (mean, SD)	7.3 (0.1)
Alveolar arterial gradient (A-a gradient) (mean, SD)	288.2 (164.3)
Pulmonary artery oxygen/FiO_2_ ratio (mean, SD)	182.5 (82.2)
Laboratory variables at 48 h after admission to ICU	
Serum calcium, mmol/L (mean, SD)	2.1 (0.1)
Blood urea nitrogen, mmol/L (mean, SD)	13.6 (8.3)
Sequestration of more than 6 L in 48 h (no. patients, %)	44 (97.8)
Blood urea nitrogen increase at least by 1.8 (no. patients, %)	26 (59.1)
PaO_2_ <60 mmHg within 48 h (no. patients, %)	12 (26.7)
Hematocrit fall >10 % (no. patients, %)	43 (95)
CCI at admission to ICU (mean, SD)	2.8 (2)
Severity of acute pancreatitis at admission to ICU	
Ranson score (mean, SD)	5.4 (1.7)
Glasgow-Imrie scale (mean, SD)	9.1 (4)
APACHE II score (mean, SD)	20.3 (6.4)
Sequential Organ Failure Assessment (mean, SD)	8.5 (3)
Abdominal compartment syndrome (no. patients, %)	16 (35.5)

*ERCP* endoscopic retrograde cholangiopancreatography, *BMI* body mass index, *LDH* lactic dehydrogenase, *AST* aspartate transaminase, *CCI* Charlson Comorbidity Index

### Obesity, morbidity, mortality, and decompressive laparotomy

Obesity was observed in 24 (53 %) patients who had clinical presentation, disease severity, and demographic characteristics similar to patients with lower BMI but significantly lower in-hospital mortality: 12.5 versus 38 % (*P* = 0.04; Table 2). Urgent DL was performed in 41 % of obese patients, whereas in only 28 % of nonobese patients (*P* = 0.533). Obese patients who underwent DL had similar clinical presentation and overall outcomes to nonobese individuals except that they experienced more postoperative incisional hernias (70 vs. 16 %; *P* = 0.039; Table 3).

**Table 2:** Characteristics of obese patients (BMI ≥ 30) versus nonobese patients

Variable	BMI ≥ 30 (*n* = 24)	BMI < 30 (*n* = 21)	*P* value
Demographic			
Age, year (mean, SD)	58.5 (13.9)	60.4 (12.8)	0.063
Male gender (no. patients, %)	19 (79.1)	17 (80.1)	0.88
Hospital mortality (no. patients, %)	3 (12.5)	8 (38.0)	**0.046**
Length of overall hospital stay, days (mean, SD)	110.7 (136.8)	57.7 (49.8)	0.087
Etiology of pancreatitis (no. patients, %)			
Gallstone	12 (50.0)	12 (57.1)	0.218
Alcohol	8 (33.3)	4 (19.0)	
Idiopathic	2 (8.3)	5 (23.8)	
Postendoscopic Cholangiopancreatography (ERCP)	2 (8.3)	0	
Use of parenteral antibiotics for prophylaxis (no. patients, %)	22 (91.6)	17 (80.9)	0.396
Clinical variables at admission to ICU			
BMI (mean, SD)	34.1 (4.3)	26.6 (2.5)	**0.0001**
Body temperature, Celsius (mean, SD)	37.9 (0.77)	37.6 (0.76)	0.168
Heart rate/min (mean, SD)	108.1 (20.2)	115.4 (17.9)	0.209
Systolic blood pressure, mmHg (mean, SD)	128.0 (17.0)	129.0 (23.8)	0.869
Diastolic blood pressure, mmHg, (mean, SD)	67.2 (12.5)	69.5 (15.1)	0.583
Respiratory rate/min (mean, SD)	19.8 (8.3)	19.7 (9.7)	0.965
Laboratory variables at admission to ICU			
White blood cells, 10^3^/μL (mean, SD)	15.1 (4.9)	14.7 (4.1)	0.327
Platelets, 10^3^/μL (mean, SD)	228.6 (177.3)	213.1 (78.6)	0.715
Creatinine, μmol/L (mean, SD)	249.6 (169.5)	181.2 (143.7)	0.155
Glucose (mmol/L) (mean, SD)	8.8 (3.4)	9.0 (5.3)	0.882
LDH, U/L (mean, SD)	521.5 (400.4)	431.1 (243.0)	0.373
AST, U/L (mean, SD)	100.3 (86.9)	79.2 (56.5)	0.348
Arterial pH (mean, SD)	7.31 (0.08)	7.35 (0.11)	0.223
Alveolar arterial gradient (A-a gradient), (mean, SD)	313.11 (180.1)	260.0 (143.1)	0.285
Pulmonary artery oxygen/FiO_2_ ratio (mean, SD)	111.5 (52.9)	95.8 (58.4)	0.35
Laboratory variables at 48 h after admission to ICU			
Serum calcium (mmol/L) (mean, SD)	2.1 (0.2)	2.1 (0.1)	0.836
Blood urea nitrogen (mmol/L) (mean, SD)	15.2 (9.4)	12.0 (6.6)	0.205
Sequestration of more than 6 L in 48 h (no. patients, %)	24 (100)	20 (95.2)	0.28
Blood urea nitrogen increase at least by 1.8 (no. patients, %)	15 (62.5)	11 (52.3)	0.387
PaO_2_ <60 mmHg within 48 h (no. patients, %)	6 (25)	6 (28.5)	0.787
Hematocrit fall >10 % (no. patients, %)	23 (95.8)	20 (95.2)	0.923
CCI at admission to ICU (mean, SD)	2.7 (1.9)	3.0 (2.1)	0.531
Severity of acute pancreatitis at admission to ICU			
Ranson score system	5.6 (1.9)	5.1 (1.5)	0.373
Glasgow scale	8.1 (4.6)	10.1 (3.0)	0.098
APACHE II score (mean, SD)	22.0 (7.0)	18.3 (5.0)	0.053
Sequential Organ Failure Assessment (mean, SD)	9.3 (3.3)	7.6 (2.5)	0.067
Decompressive laparotomy (no. patient, %)	10 (41.6)	6 (28.5)	0.533

Bold indicate statistical significant values

*ERCP* endoscopic retrograde cholangiopancreatography, *BMI* body mass index, *LDH* lactic dehydrogenase, *AST* aspartate transaminase, *ICU* intensive care unit

**Table 3:** Summary of clinical characteristics, early and late management of the open abdomen after decompressive laparotomy in obese (BMI ≥ 30) and nonobese patients

Variable	BMI ≥ 30 (*n* = 10)	BMI < 30 (*n* = 6)	*P* value
Intra-abdominal pressure, mmHg (bladder pressure measurement, mean, SD)	26.7 (9.3)	32.3 (15.2)	0.361
Patients with bladder pressure ≥20 mmHg (no. patients, %)	8 (80)	5 (83.3)	0.482
Clinical presentation of ACS (no. patients, %)			
Abdominal distension with acute renal failure	4 (40)	1 (16.6)	0.588
Abdominal distension with acute respiratory failure	6 (60)	5 (83.3)	
Time between diagnosis of ACS and surgical decompression, hr (mean, SD)	3.3 (1.7)	2.8 (1.7)	0.638
Early management of abdominal incision (no. patients, %)			
Bogota bag	7 (70)	4 (66.6)	0.889
Wound VAC system	3 (30)	2 (33.3)	
Late management of abdominal incision (no. patients, %)			
Delayed primary abdominal wall closure	6 (60)	5 (83.3)	0.33
Use of split-thickness skin graft	3 (30)	0	0.137
Death before abdominal incision closure	1 (10)	1 (16.6)	0.761
Abdominal complication after decompressive laparotomy (no. patients, %)			
Pancreatico-cutaneous fistula	1 (10)	2 (33.3)	0.247
Entero-cutaneous / entero-atmospheric fistula	5 (50)	2 (33.3)	0.515
Incisional infection	8 (80)	2 (33.3)	0.062
Wound dehiscence	3 (30)	0	0.137
Incisional hernia	7 (70)	1 (16.6)	**0.039**
In-hospital mortality (no. patients, %)	1 (10)	2 (33.3)	0.252

Bold indicate statistical significant values

### In-hospital mortality

Among all 45 patients, 11 (24.4 %) died from complications of SAP (Table 4). Univariate analysis showed that mortality was associated with older age (*P* = 0.001), lower systolic blood pressure at admission to ICU (*P* = 0.05), and higher number of comorbidities (*P* = 0.001). Predicted mortality by Ranson criteria and by APACHE II score for the entire cohort were 41 % (SD = 30.2) and 38.3 % (SD = 19.4), respectively. Comparison between observed (24 %) and predicted (38–41 %) mortality by APACHE II Score of the entire cohort was clinically but not statistically significant (*P* = 0.175).

**Table 4:** Characteristics of patients who died versus patients who survived severe acute pancreatitis

Variable	Patients who died (*n* = 11)	Patients who survived (*n* = 34)	*P* value
Demographic			
Age, year (mean, SD)	70.9 (11.9)	55.7 (11.6)	**0.001**
Male gender (no. patients, %)	9 (81.8)	27 (79.4)	0.78
Etiology of pancreatitis (no. patients, %)			
Gallstone	9 (81.8)	15 (44.1)	0.176
Alcohol	1 (9)	11 (32.3)	
Idiopathic	1 (9)	6 (17.6)	
Postendoscopic cholangiopancreatography (ERCP)	0	2 (5.8)	
Length of overall hospital stay, days (mean, SD)	25 (21.9)	105.7 (117.1)	**0.017**
Clinical variables on admission to ICU			
BMI (mean, SD)	28.6 (3.1)	31.2 (5.6)	0.128
Obesity (BMI > 30) (no. patients, %)	3 (27.2)	21 (61.7)	0.08
Body temperature, Celsius (mean, SD)	37.5 (0.7)	37.8 (0.7)	0.199
Heart rate/min (mean, SD)	112.0 (19.2)	111.4 (19.6)	0.921
Systolic blood pressure, mmHg (mean, SD)	117.6 (19.3)	131.8 (19.4)	**0.05**
Diastolic blood pressure, mmHg (mean, SD)	64.7 (9)	69.4 (14.7)	0.348
Respiratory rate/min (mean, SD)	20.7 (10.6)	19.4 (8)	0.68
Bladder pressure measurement, mmHg (mean, SD)	22.7 (5.1)	30.8 (12.7)	0.24
Laboratory variables on admission to ICU			
White blood cells, 10^3^/μL (mean, SD)	13.7 (3.7)	15.3 (4.7)	0.31
Platelets, 10^3^/μL (mean, SD)	217.5 (80.1)	222.6 (154.2)	0.91
Creatinine, μmol/L (mean, SD)	211.0 (126.4)	219.9 (171)	0.87
Glucose, mmol/L (mean, SD)	11.0 (6.9)	8.3 (3)	0.069
LDH, U/L (mean, SD)	326.3 (113.4)	528.8 (368.9)	0.082
AST, U/L (mean, SD)	67.2 (34.8)	98.0 (82.1)	0.237
Arterial pH (mean, SD)	7.30 (0.92)	7.34 (0.1)	0.354
Alveolar arterial gradient (A-a gradient) (mean, SD)	261.1 (105.3)	297.1 (179.7)	0.531
Pulmonary artery oxygen/FiO_2_ ratio (mean, SD)	117.7 (58.9)	99.7 (54.5)	0.357
Laboratory variables at 48 h after admission to ICU			
Serum calcium, mmol/L (mean, SD)	2.1 (0.2)	2.1 (0,.17)	0.64
Blood urea nitrogen, mmol/L (mean, SD)	12.5 (2.4)	14.0 (9.3)	0.641
Sequestration of more than 6 L in 48 h (no. patients, %)	11 (100)	33 (97)	1
Blood urea nitrogen increase at least by 1.8 (no. patients, %)	7 (63.6)	19 (55.8)	0.489
PaO_2_ <60 mmHg within 48 h (no. patients, %)	3 (27.2)	9 (26.4)	1
Hematocrit fall >10 % (no. patients, %)	3 (27.2)	9 (26.4)	1
CCI at admission to ICU (mean, SD)	2.3 (1.7)	4.5 (2)	**0.001**
Severity of acute pancreatitis on admission to ICU			
Ranson score system	5.63 (1.9)	5.38 (1.9)	0.683
Glasgow scale	10.1 (1.9)	8.7 (4.5)	0.324
APACHE II score (mean, SD)	20.6 (4.6)	20.2 (6.9)	0.87
Sequential Organ Failure Assessment (mean, SD)	8.6 (2.2)	8.5 (3.3)	0.921
Decompressive laparotomy (no. patients, %)	4 (36.3)	12 (35.2)	1.00
Early management of abdominal incision (no. patients, %)			
Bogota bag	2 (18.1)	9 (26.4)	0.547
Wound VAC system	2 (18.1)	3 (8.8)	
Late management of abdominal incision (no. patients, %)			
Delayed primary abdominal wall closure	3 (27.2)	8 (23.5)	1
Use of split-thickness skin graft	0	3 (8.8)	0.529
Complication after abdominal wall closure (no. patients, %)			
Pancreatico-cutaneous fistula	0	3 (8.8)	0.529
Entero-cutaneous fistula	1 (9.0)	6 (17.6)	0.585
Incisional infection	1	9 (26.4)	0.118
Wound dehiscence	1	2 (5.8)	1
Incisional hernia	1	7 (20.5)	0.569

Bold indicate statistical significant values

*ERCP* endoscopic retrograde cholangiopancreatography, *BMI* body mass index, *LDH* lactic dehydrogenase, *AST* aspartate transaminase, *ICU* intensive care unit, *CCI* Charlson Comorbidity Index

Univariate logistic regression analysis showed that age was a significant predictor of in-hospital mortality for the entire cohort (odds ratio (OR) 1.159; 95 % CI = 1.043–1.288; *P* = 0.006) and more so for obese patients (OR 1.191; 95 % CI = 1.012–1.401; *P* = 0.035; Table 5). After adjusting for the severity of the disease by Ranson, SOFA, APACHE II and Glasgow-Imrie scores, obesity status, and development of ACS, the only significant predictor for in-hospital mortality in the study population was patients’ age (OR 1.273; 95 % CI = 1.052–1.541; *P* = 0.013).

**Table 5:** Univariate logistic regression: risk factors for in-hospital mortality

Variable	OR for entire study population (95 % CI)	*P* value	OR for obese patients (95 % CI)	*P* value	OR for nonobese patients (95% CI)	*P* value
Age (year)	1.159 (1.043, 1.288)	**0.006**	1.191 (1.012, 1.401)	**0.035**	1.134 (0.99, 1.298)	0.069
Systolic blood pressure (mmHg)	0.956 (0.913, 1.002)	0.058	0.966 (0.895, 1.044)	0.966	0.051 (0.896, 1.01)	0.103
Obesity (BMI > 30)	4.308 (0.965, 19.236)	0.056	**–**	**–**	**–**	**–**
Abdominal compartment syndrome	0.955 (0.232, 3.932)	0.949	**–**	**–**	**–**	**–**
Charlson Comorbidity score	1.845 (1.199, 2.84)	**0.005**	3.782 (1.06, 13.489)	**0.04**	1.518 (0.929, 2.483)	0.096
Ranson score	1.085 (0.749, 1.591)	0.676	0.789 (0.376, 1.654)	0.53	1.761 (0.872, 3.555)	0.114
SOFA score	1.012 (0.808, 1.267)	0.318	1.164 (0.782, 1.733)	0.454	1.057 (0.739, 1.513)	0.761
APACHE II score	1.009 (0.907, 1.124)	0.866	1.06 (0.875, 1.283)	0.553	1.061 (0.882, 1.277)	0.529
Glasgow-Imrie score	1.095 (0.917, 1.307)	0.919	1.106 (0.842, 1.453)	0.47	1.011 (0.75, 1.362)	0.942

Bold indicate statistical significant values

**Fig. 1:**
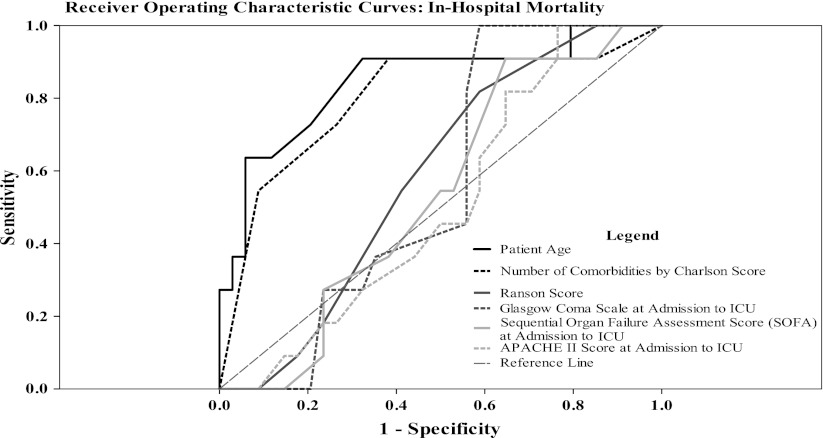
Receiver operating characteristic (ROC) curves for hospital mortality of patients affected by severe acute pancreatitis (SAP). Area under the curve (AUC) for discriminating variables at the time of patients’ admission to the intensive care unit (ICU). **a** *Patients’ age* = 0.846 (95 % confidence interval (CI) = 0.7–0.99; *P* = 0.001), **b** *Charlson Comorbidity Score* = 0.801 (95 % CI = 0.633–0.968; *P* = 0.003), **c** *Ranson criteria* = 0.584 (95 % CI = 0.415–0.753; *P* = 0.405), **d** *Glasgow Coma Scale* = 0.56 (95 % CI = 0.394–0.726; *P* = 0.552), **e** *Sequential Organ Failure Assessment Score (SOFA)* = 0.537 (95 % CI = 0.363–0.712; *P* = 0.712), **f** *Acute Physiologic and Chronic Health Evaluation (APACHE) II score* = 0.509 (95 % CI = 0.334–0.684; *P* = 0.926) (All *P* values are calculated for each variable in comparison to the reference line; AUC = 0.5)

Receiver operator characteristic (ROC) curves for in-hospital mortality for ICU patients with SAP showed that both age and the number of comorbidities had excellent discrimination with AUC measuring 0.846 (95 % CI = 0.7–0.99; *P* = 0.001) and 0.801 (95 % CI = 0.633–0.968; *P* = 0.003), respectively (Fig. 1). On the other hand, established predictive models (Ranson, Glasgow-Imrie, SOFA, APACHE II) had low discrimination with AUC ranging from 0.5 (APACHE II) to 0.584 (Ranson).

#### Risk factors for abdominal compartment syndrome and outcomes of decompressive laparotomy

Analysis of the clinical and laboratory characteristics of patient who developed ACS and underwent DL revealed that they were more frequently males (*P* = 0.03), had a higher respiratory and heart rate (*P* = 0.008, 0.01), lower systolic blood pressure (*P* = 0.05), and higher serum creatinine levels (*P* = 0.04) at admission to ICU than patients who did not develop ACS (Table 6). Univariate logistic regression analysis found that only serum creatinine was associated with higher probability of developing ACS (OR 1.115; 95 % CI = 1.02–1.219; *P* = 0.017; Table 7). At multivariable regression analysis, after adjusting for the severity of the disease (Ranson, SOFA, APACHE II, Glasgow-Imrie Scores), age, and presence of obesity, only Glasgow-Imrie and APACHE II scores were significant predictors for ACS with OR of 1.221 and 1.143, respectively (Glasgow-Imrie 95 % CI = 1.000–1.493; *P* = 0.05) (APACHE II 95 % CI = 1.012–1.292; *P* = 0.032).

**Table 6:** Characteristics of patients requiring decompressive laparotomy versus patients who did not undergo decompressive laparotomy

Variable	Decompressive laparotomy (*n* = 16)	Nondecompressive laparotomy (*n* = 29)	*P* value
Demographic			
Age, year (mean, SD)	55.9 (12.9)	60.7 (14)	0.39
Male gender (no. patients, %)	16 (100)	20 (68.9)	**0.03**
Etiology of pancreatitis (no. patients, %)			
Gallstone	7 (43.8)	17 (58.6)	0.22
Alcohol	7 (43.8)	6 (20.6)	
Idiopathic	2 (12.5)	5 (17.2)	
Postendoscopic Cholangiopancreatography (ERCP)	0	2 (6.8)	
Length of overall hospital stay, days (mean, SD)	146.2 (148.5)	60.9 (65.2)	**0.001**
Clinical variables at admission to ICU			
BMI (mean, SD)	30.3 (3.7)	30.8 (5.8)	0.66
Obesity (BMI > 30) (no. patients, %)	10 (62.5)	15 (51.7)	0.48
Body temperature, Celsius (mean, SD)	37.7 (0.8)	37.8 (0.7)	0.82
Heart rate/min (mean, SD)	122.5 (14.3)	107.1 (19.4)	**0.01**
Systolic blood pressure, mmHg (mean, SD)	119.4 (21.3)	132.5 (18.5)	**0.05**
Diastolic blood pressure, mmHg (mean, SD)	65.4 (15.9)	69.5 (12.5)	0.37
Respiratory rate/min (mean, SD)	27.0 (11.4)	16.8 (5.4)	**0.008**
Laboratory variables at admission to ICU			
White blood cells, 10^3^/μL (mean, SD)	17.7 (5.1)	13.8 (5)	0.29
Platelets, 10^3^/μL (mean, SD)	187.3 (90.6)	235.2 (153.4)	0.29
Creatinine, μmol/L (mean, SD)	292.6 (196.3)	187.2 (134.5)	**0.04**
Glucose, mmol/L (mean, SD)	10.7 (3.5)	8.2 (4.5)	0.08
LDH, U/L (mean, SD)	485.0 (348.2)	477 (336.3)	0.94
AST, U/L (mean, SD)	73.2 (36.6)	97.5 (84.5)	0.32
Arterial pH (mean, SD)	7.2 (0.9)	7.3 (0.1)	0.11
Alveolar arterial gradient (A-a gradient) (mean, SD)	346.2 (167.2)	264.8 (159.7)	0.13
Pulmonary artery oxygen/FiO_2_ ratio (mean, SD)	91.3 (25.5)	109.3 (63.4)	0.33
Laboratory variables at 48 h after admission to ICU			
Serum calcium, mmol/L (mean, SD)	2.1 (0.1)	2.2 (0.1)	0.2
Blood urea nitrogen, mmol/L (mean, SD)	13.5 (8.3)	13.7 (8.4)	0.95
Sequestration of more than 6 L in 48 h (no. patients, %)	16 (100)	28 (96.5)	0.51
Blood urea nitrogen increase at least by 1.8 (no. patients, %)	9 (56.2)	17 (58.6)	0.74
PaO_2_ <60 mmHg within 48 h (no. patients, %)	4 (25)	9 (31)	0.46
Hematocrit fall >10 % (no. patients, %)	16 (100)	26 (89.6)	0.3
CCI at admission to ICU (mean, SD)	2 (1.6)	3.2 (2)	0.06
Severity of acute pancreatitis at admission to ICU			
Ranson score system	6 (1.8)	5.2 (1.6)	0.18
Glasgow scale	9.9 (4.7)	8.7 (4.6)	0.4
APACHE II score (mean, SD)	22.6 (6.2)	19.4 (6.3)	0.13
Sequential Organ Failure Assessment (mean, SD)	9 (2.9)	8.3 (3.1)	0.47
Time interval between diagnosis of ACS and DL (h) (mean, SD)	3.1 (1.7)	n.a.	–
In-hospital mortality (no. patients, %)	4 (25)	7 (24.1)	0.9

Bold indicate statistical significant values

*ERCP* endoscopic retrograde cholangiopancreatography, *BMI* body mass index, *LDH* lactic dehydrogenase, *AST* aspartate transaminase, *ICU* intensive care unit, *DL* decompressive laparotomy, *ACS* abdominal compartment syndrome, *n.a.* not applicable

**Table 7:** Univariate logistic regression: risk factors for abdominal compartment syndrome

Variable	OR for entire study population (95 % Cl)	*P* value	OR for obese patients (95% CI)	*P* value	OR for nonobese patients (95 % CI)	*P* value
Serum creatinine (μmol/L)	1.115 (1.02, 1.219)	**0.017**	1.005 (0.999, 1.01)	0.103	1.003 (0.996, 1.009)	0.412
Respiratory Rate/minute	1.004 (1, 1.008)	0.053	1.071 (0.961, 1.193)	0.213	1.206 (0.997, 1.46)	0.054
Age	0.968 (0.922, 1.017)	0.195	0.961 (0.899, 1.028)	0.249	0.981 (0.911, 1.055)	0.597
Obesity (BMI > 30)	0.56 (0.161, 1.949)	0.362	**–**	**–**	**–**	**–**
Charlson Comorbidity score	0.79 (0.568, 1.099)	0.162	0.951 (0.622, 1.453)	0.816	0.603 (0.327, 1.114)	0.106
Ranson score	1.13 (0.798, 1.601)	0.49	1.166 (0.763, 1.783)	0.478	0.985 (0.516, 1.878)	0.963
SOFA score	1.079 (0.88, 1.322)	0.465	1.062 (0.825, 1.369)	0.64	1.039 (0.708, 1.525)	0.844
APACHE II score	1.085 (0.978, 1.204)	0.122	1.05 (0.929, 1.185)	0.436	1.163 (0.924, 1.464)	0.197
Glasgow-Imrie score	1.064 (0.911, 1.241)	0.434	1.148 (0.95, 1.388)	0.152	0.92 (0.667, 1.268)	0.61

Bold indicate statistical significant values

Hospital stay for patients who underwent DL was significantly longer (146 vs. 60 days; *P* = 0.001) compared with patients who did not develop ACS, but they did not experience higher mortality rates (25 vs. 24 %; *P* = 0.9). All 16 patients who underwent DL had a temporary abdominal wall closure with either Bogota bag (11 subjects) or wound V.A.C system^®^ (five subjects). Delayed primary closure was performed in 11 patients and split-thickness skin graft was necessary in five. Perioperative complications of DL were significant with ten surgical site infections that ultimately led to eight hernias requiring delayed repair, seven entero-atmospheric fistulas that were managed by late intestinal resections, three wound dehiscences that were fixed surgically, and three pancreatico-atmospheric fistulas that resolved without any further surgical intervention.

**Fig. 2:**
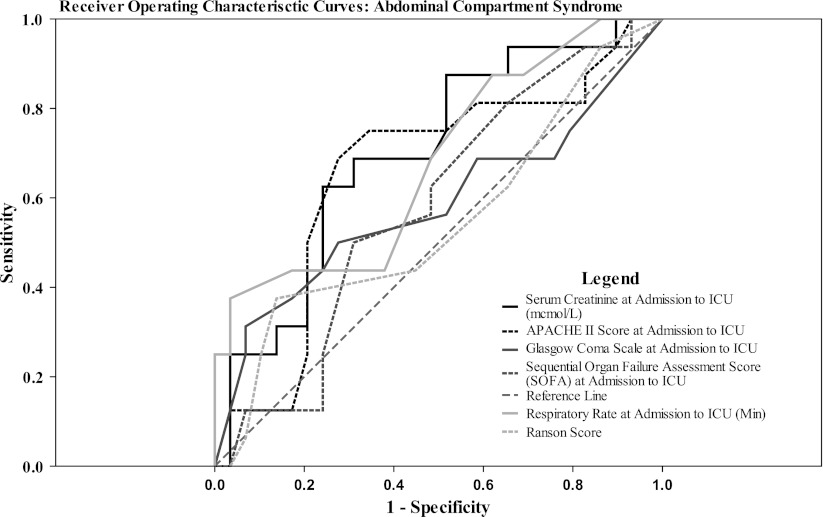
Receiver operating characteristic (ROC) curves for the development of abdominal compartment syndrome (ACS) in patients affected by severe acute pancreatitis (SAP). Area under the curve (AUC) for discriminating variables at the time of patients’ admission to the intensive care unit (ICU), **a** *Serum creatinine* = 0.699 (95 % CI = 0.54–0.858; *P* = 0.028), **b** *Respiratory rate* = 0.681 (95 % CI = 0.515–0.847; *P* = 0.046), **c** Acute Physiologic and Chronic Health Evaluation (*APACHE*) *II score* = 0.661 (95 % CI = 0.487–0.835; *P* = 0.07, **d** *Glasgow Coma Scale* = 0.584 (95 % CI = 0.394–0.774; *P* = 0.355), **e** *Sequential Organ Failure Assessment Score* (*SOFA*) = 0.582 (95 % CI = 0.411–0.753; *P* = 0.368), **f** Obesity (BMI ≥ 30) = 0.571 (95 % CI = 0.396–0.747; *P* = 0.434), **g** *Ranson score* = 0.548 (95 % CI = 0.365–0.732; *P* = 0.594) (All *P* values are calculated for each variable in comparison to the reference line; AUC = 0.5)

ROC curves for the development of ACS showed that there were no clinical or laboratory characteristics with acceptable discrimination (Fig. 2). Serum creatinine level at admission to ICU and respiratory rate had better discrimination performance with AUC equal to 0.69 and 0.68, respectively, compared with established prognostic models with AUC ranging from 0.58 (Ranson) to 0.66 (APACHE II). Presence of obesity had poor discrimination with AUC = 0.57.

## Discussion

### Obesity, mortality, development of abdominal compartment syndrome and decompressive laparotomy

To our knowledge, this is the first study to compare the outcomes of obese patients admitted to the ICU with SAP versus nonobese individuals. Other authors published outcomes from retrospective cohorts of all patients affected by AP and did not restrict their analysis to those requiring ICU care for SAP [19–21, 26, 54–59]. Our study has shown that, in these settings, obesity might be protective for mortality but not for the development of ACS. Because obese patients underwent DL more often than nonobese individuals, their lower mortality is quite provocative, because it seems to conflict with previous studies [19–21, 24, 26, 54–59]. Lankisch et al. [60] were the first to report higher risk of respiratory, renal, and circulatory insufficiency in patients with elevated body weight affected by AP. Several other small studies have subsequently reported a positive correlation between obesity and respiratory failure, local complications, and death [20, 54, 55]. A recent meta-analysis found a twofold increase in mortality in obese patients with AP [21]. The mechanisms by which obesity adversely affects the course of AP are still unclear [24, 56]. Overweight patients have an up-regulated systemic [53, 54, 58, 61] and local inflammatory response [24, 52, 57, 62]. They are more frequently immunodeficient [61, 62] and have larger deposits of retroperitoneal and visceral fat where necrosis and infections frequently occur in AP [24, 52, 63, 64]. Their pancreatic microcirculation is to some extent compromised by fat deposition in the gland, and the pancreas therefore is more prone to necrosis [26]. Excessive body weight also reduces the compliance of the chest wall, diaphragm, and abdominal wall with subsequent increased thoracic and peritoneal pressures [26]. The negative prognostic factor represented by obesity is therefore supported by both biological and clinical observations [24]. However, there is still controversy on this topic [1, 2] as the evidence that obese patients experience worse outcome [19, 21, 54–56] has been challenged by several more recent large clinical studies [22, 25, 59]. Mery et al. [61] have shown that the distribution of fat in the body might be more important than obesity itself for the modulation of SIRS and the risk of mortality in AP. Recent experimental data suggested that visceral fat produces more inflammatory mediators than subcutaneous fat [62], and a clinical study has found that only increased visceral fat distribution was a negative predictor of survival in AP [61].

### Prediction of mortality

In our cohort, the observed mortality was 24 % and not significantly different from other studies that reported a range between 17 and 39 % [65–67]. Compared with the predicted mortality by Ranson and the APACHE II scores, the observed death rate was much lower, although it did not reach statistical significance. There are many possible reasons for the discrepancy between predicted and observed mortality in this study. During the past few decades, significant improvements occurred in the management of MOF patients with SAP [1], including the introduction of parenteral and enteral nutrition and more effective broad-spectrum antibiotics [1, 68]. Surgeons also have played an important role in improving the overall outcomes of patients with SAP by being more selective when recommending surgery and by using less invasive procedures for the debridement of necrotic tissue [1, 63]. As a result, a growing proportion of patients with SAP is now able to overcome both the early systemic inflammatory response syndrome (SIRS) and the second phase of illness characterized by sepsis and organ failure [57, 64, 69–71]. Because of these important changes in the management of patients with SAP, it might be necessary to reevaluate the role of predicting models that were introduced several decades ago [5–9, 23, 24, 28–30, 32, 65, 66, 72]. In our study, advanced age and the presence of comorbidities seemed to play a more influential role than any of the well recognized predicting models. Contrary to the results reported by Ueda et al. [67] who observed that both Ranson and APACHE II scores had good discrimination for mortality with AUC of 0.8, our findings supported the results of other authors [58, 68, 73], who observed that Ranson score was a relatively poor predictor for in-hospital mortality, whereas age and the presence of comorbidities were better discriminating factors. While the limited sample size may account for some variations, it also is possible that different inclusion criteria might have played an important role as other studies did not include only subjects affected by SAP and requiring ICU admission [69].

### Prediction of abdominal compartment syndrome

ACS was observed in 35 % of this cohort, similarly to previous studies where 23 [74] and 56 % [75] of patients with SAP developed IAH. The clinical relevance of ACS is illustrated by the significantly longer hospital stay compared with those who did not require DL as previously described by Al-Bahrani et al. [75]. Contrary to our experience, De Waele et al. [76] not only reported significantly longer ICU and hospital stays in patients with IAH but also an increased mortality. In our study, the mortality rate of patients who developed ACS was 25 %, similar to that observed in patients who did not develop IAH. This might be due to the fact that patients who underwent DL at our institution were taken to the operative room within a very short period as the median time interval from diagnosis to the start of DL was only 3.1 h. The rationale to perform an urgent DL was to restore, as soon as possible, the physiological microcirculatory parameters and avoid further damage to the renal and cardiorespiratory systems [77]. Although this might be valid for trauma patients, we recognize that there is lack of studies to support this strategy in AP and it is still unknown the optimal time for intervention and the optimal threshold of IAP that would mandate surgical decompression in patients with SAP [4, 78, 79]. It also is unclear the degree to which IAH contributes to the progression of organ dysfunction in patients with SAP and deteriorating organ function. Mentula et al. [77] found that patients with IAP exceeding 25 mmHg within the first 4 days after diagnosis of SAP might be good candidates for surgical decompression, but there is need for more studies to support these findings.

### Outcomes of decompressive laparotomy

Substantial morbidity was associated with DL, and the decision to perform surgery had important clinical implications [80]. An open abdomen is a well-recognized risk factor for the development of fistulas, intra-abdominal abscesses, abdominal wall infections, and hernias whose management can be very challenging especially in obese patients [80]. In this study, enterocutaneous or enteroatmospheric fistulas occurred in 43 % of the subjects undergoing DL, 18 % required the use of split-thickness skin graft for the closure of their incision, and 50 % developed incisional hernias that required delayed repair [81]. Overall, 85 % of patients undergoing DL needed at least another surgical intervention for the management of their open abdomen, and compared with patients treated by conservative measures and who survived, their hospital stay was approximately 2 months longer.

### Predictive models

Contrary to other authors [41, 76, 77], we did not identify any significant difference between the values of predicting scores in patients who underwent DL and the group treated conservatively. We also failed to identify any variable that could classify patients at risk of developing ACS as the discriminating performance of all the severity scoring systems, such as APACHE II, SOFA, Ranson, Glasgow-Imrie, and other predictors, including BMI had ROC curves with AUC ranging only between 0.54 and 0.69. Dambrauskas et al. [41] observed good discriminating characteristics for APACHE II (AUC of 0.86) and for Glasgow-Imrie score (AUC of 0.92). In their study, APACHE II cutoff of seven had a sensitivity of 100 % and specificity of 60 % for the diagnosis of ACS. Similarly, Glasgow-Imrie score greater than three had a sensitivity of 83 % and specificity of 86 %. Several factors may explain these variations as the inclusion and exclusion criteria were different and the proportion of patients with obesity was not specified. Therefore, interpreting and comparing the results is difficult considering the heterogeneity of patient populations, methods, and outcome reporting among all the various publications [11].

### Strengths and limitations

One of the strengths of this study is that it is the largest experience of patients affected by SAP as defined by the Atlanta consensus conference and admitted in ICU at a tertiary teaching center. Another important aspect is that strict inclusion and exclusion criteria were applied and that a power calculation was performed to assess the number of subjects needed to identify possible differences for the primary outcome between groups. Lastly, established and clear definitions were used for the description of the study population, clinical investigations, and surgical interventions necessary for comparison with other studies.

Our study has several limitations due to its retrospective design. During the time period of this study, there were no protocols or established algorithms specific to our institution for the management of patients affected by SAP. This led to variations on the decision-making process among all the physicians practicing in our ICU. As a consequence, SAP patients did not undergo routine measurements of their abdominal pressure unless they manifested deterioration of their cardiopulmonary or renal function. The decision to perform DL also was made selectively by the surgeon staff on call once notified of the clinical deterioration of the patient or the elevated value of the intra-abdominal pressure after conservative measures, such as the use of paralyzing agents and/or aggressive diuresis failed [39]. Because there is still no uniform consensus in the literature on the indications for surgical decompression in ACS associated with SAP [40, 74], it is very likely that in many circumstances the value of intra-abdominal pressure was not the only parameter used for the decision to perform a DL. It is possible that in this cohort some patients might have developed ACS, which likely a rare event because the overall mortality of our study population was comparable to that reported by other investigators who recommend routine measurement of the intra-abdominal pressures in all patients admitted in ICU for SAP [59, 82].

## Conclusions

Mortality of patients with SAP has declined significantly during the past few decades despite the increasing number of obese patients in the general population. This study suggests that obesity per se is not a negative prognostic factor for mortality in patients with SAP admitted to the ICU. DL is associated with higher rates of fistulization and required multiple surgical interventions for the management of the open abdomen. Nevertheless, mortality rate of patients undergoing DL was similar to patients who did not require surgery suggesting that, in selected patients, DL might have a significant role in reversing the declining course of SAP. Because most of the current literature is retrospective and from the experience of single-centers with limited number of patients, prospective studies are necessary to assess the impact of obesity, fat distribution, and other variables, such as age or comorbidities, on mortality and morbidity of patients with SAP requiring ICU admission.

## References

[1] Bradley EL, Dexter ND (2010). Management of severe acute pancreatitis: a surgical odyssey. Ann Surg.

[2] Corfield AP, Cooper MJ, Williamson RC (1985). Acute pancreatitis: a lethal disease of increasing incidence. Gut.

[3] Mann DV, Hershman MJ, Hittinger R, Glazer G (1994). Multicentre audit of death from acute pancreatitis. Br J Surg.

[4] McKay CJ, Evans S, Sinclair M, Carter CR, Imrie CW (1999). High early mortality rate from acute pancreatitis in Scotland, 1984–1995. Br J Surg.

[5] Ranson JH, Rifkind KM, Roses DF, Fink SD, Eng K, Spencer FC (1974). Prognostic signs and the role of operative management in acute pancreatitis. Surg Gynecol Obstet.

[6] Imrie CW, Benjamin IS, Ferguson JC, McKay AJ, Mackenzie I, O’Neill J (1978). A single-centre double-blind trial of Trasylol therapy in primary acute pancreatitis. Br J Surg.

[7] Knaus WA, Zimmerman JE, Wagner DP, Draper EA, Lawrence DE (1981). APACHE-acute physiology and chronic health evaluation: a physiologically based classification system. Crit Care Med.

[8] Vincent JL, Moreno R, Takala J, Willatts S, De Mendonça A, Bruining H (1996). The SOFA (Sepsis-related Organ Failure Assessment) score to describe organ dysfunction/failure. On behalf of the Working Group on Sepsis-Related Problems of the European Society of Intensive Care Medicine. Intensive Care Med.

[9] Spitzer AL, Barcia AM, Schell MT, Barber A, Norman J, Grendell J (2006). Applying Ockham’s Razor to pancreatitis prognostication: a four-variable predictive model. Ann Surg.

[10] Tenner S, Sica G, Hughes M, Noordhoek E, Feng S, Zinner M (1997). Relationship of necrosis to organ failure in severe acute pancreatitis. Gastroenterology.

[11] Deenadayalu VP, Blaut U, Watkins JL, Barnett J, Freeman M, Geenen J (2008). Does obesity confer an increased risk and/or more severe course of post-ERCP pancreatitis? A retrospective, multicenter study. J Clin Gastroenterol.

[12] Yanovski SZ, Yanovski JA (2011). Obesity prevalence in the United States–up, down, or sideways?. N Engl J Med.

[13] Pajuelo-Ramírez J, Miranda-Cuadros M, Campos-Sánchez M, Sánchez-Abanto J (2011). Prevalence of overwight and obesity among children under five years in Peru 2007–2010. Rev Peru Med Exp Salud Publica.

[14] Oh I-H, Cho Y, Park S-Y, Oh C, Choe B-K, Choi J-M (2011). Relationship between socioeconomic variables and obesity in Korean adolescents. J Epidemiol.

[15] Misra A, Khurana L (2011). Obesity-related non-communicable diseases: South Asians vs white Caucasians. Int J Obes.

[16] Misra A, Shah P, Goel K, Hazra DK, Gupta R, Seth P (2011). The high burden of obesity and abdominal obesity in urban Indian schoolchildren: a multicentric study of 38,296 children. Ann Nutr Metab.

[17] Calle EE, Rodriguez C, Walker-Thurmond K, Thun MJ (2003). Overweight, obesity, and mortality from cancer in a prospectively studied cohort of U.S. adults. N Engl J Med.

[18] Márquez-Sandoval F, Macedo-Ojeda G, Viramontes-Hörner D, Fernández Ballart JD, Salas Salvadó J, Vizmanos B (2011). The prevalence of metabolic syndrome in Latin America: a systematic review. Public Health Nutr.

[19] Funnell IC, Bornman PC, Weakley SP, Terblanche J, Marks IN (1993). Obesity: an important prognostic factor in acute pancreatitis. Br J Surg.

[20] Martínez J, Sánchez-Payá J, Palazón JM, Aparicio JR, Picó A, Pérez-Mateo M (1999). Obesity: a prognostic factor of severity in acute pancreatitis. Pancreas.

[21] Martínez J, Sánchez-Payá J, Palazón JM, Suazo-Barahona J, Robles-Díaz G, Pérez-Mateo M (2004). Is obesity a risk factor in acute pancreatitis? A meta-analysis. Pancreatology.

[22] De Waele B, Vanmierlo B, Van Nieuwenhove Y, Delvaux G (2006). Impact of body overweight and class I, II and III obesity on the outcome of acute biliary pancreatitis. Pancreas.

[23] Halonen KI, Leppaniemi AK, Puolakkainen PA, Lundin JE, Kemppainen EA, Hietaranta AJ (2000). Severe acute pancreatitis: prognostic factors in 270 consecutive patients. Pancreas.

[24] Papachristou GI, Papachristou DJ, Avula H, Slivka A, Whitcomb DC (2006). Obesity increases the severity of acute pancreatitis: performance of APACHE-O score and correlation with the inflammatory response. Pancreatology.

[25] Tsai CJ (1998). Is obesity a significant prognostic factor in acute pancreatitis?. Dig Dis Sci.

[26] Frossard J-L, Lescuyer P, Pastor CM (2009). Experimental evidence of obesity as a risk factor for severe acute pancreatitis. World J Gastroenterol.

[27] Suazo-Barahona J, Carmona-Sánchez R, Robles-Díaz G, Milke-García P, Vargas-Vorácková F, Uscanga-Domínguez L (1998). Obesity: a risk factor for severe acute biliary and alcoholic pancreatitis. Am J Gastroenterol.

[28] Larvin M, McMahon MJ (1989). APACHE-II score for assessment and monitoring of acute pancreatitis. Lancet.

[29] Knaus WA, Wagner DP, Draper EA, Zimmerman JE, Bergner M, Bastos PG (1991). The APACHE III prognostic system. Risk prediction of hospital mortality for critically ill hospitalized adults. Chest.

[30] Ranson JH, Rifkind KM, Roses DF, Fink SD, Eng K, Localio SA (1974). Objective early identification of severe acute pancreatitis. Am J Gastroenterol.

[31] Charlson ME, Pompei P, Ales KL, MacKenzie CR (1987). A new method of classifying prognostic comorbidity in longitudinal studies: development and validation. J Chronic Dis.

[32] Juneja D, Gopal PB, Ravula M (2010). Scoring systems in acute pancreatitis: which one to use in intensive care units?. J Crit Care.

[33] MedCalc (2011) http://www.mdcalc.com/ransons-criteria-for-pancreatitis-mortality/. Accessed 11 Nov 2011

[34] Knaus WA, Draper EA, Wagner DP, Zimmerman JE (1985). APACHE II: a severity of disease classification system. Crit Care Med.

[35] Bradley EL (1993). A clinically based classification system for acute pancreatitis. Summary of the international symposium on acute pancreatitis, Atlanta, GA, September 11–13, 1992. Arch Surg.

[36] Group APCW (2011) Revision of the Atlanta classification of acute pancreatitis. http://pancreasclub.com/wp-content/uploads/2011/11/AtlantaClassification.pdf. Accessed 10 Nov 2011

[37] Venkatesan T, Moulton JS, Ulrich CD, Martin SP (2003). Prevalence and predictors of severity as defined by Atlanta criteria among patients presenting with acute pancreatitis. Pancreas.

[38] SFAR Web Page (2012) http://www.sfar.org/scores2/apache22.html. Accessed 13 Jan 2012

[39] Cheatham ML, Malbrain MLNG, Kirkpatrick A, Sugrue M, Parr M, De Waele J (2007). Results from the international conference of experts on intra-abdominal hypertension and abdominal compartment syndrome. II. Recommendations. Intensive Care Med.

[40] Tao J, Wang C, Chen L, Yang Z, Xu Y, Xiong J (2003). Diagnosis and management of severe acute pancreatitis complicated with abdominal compartment syndrome. J Huazhong Univ Sci Technol Med Sci.

[41] Dambrauskas Z, Parseliunas A, Gulbinas A, Pundzius J, Barauskas G (2009). Early recognition of abdominal compartment syndrome in patients with acute pancreatitis. World J Gastroenterol.

[42] Radenkovic DV, Bajec D, Ivancevic N, Bumbasirevic V, Milic N, Jeremic V (2010). Decompressive laparotomy with temporary abdominal closure versus percutaneous puncture with placement of abdominal catheter in patients with abdominal compartment syndrome during acute pancreatitis: background and design of multicenter, randomised, controlled study. BMC Surg.

[43] Leppäniemi AK, Hienonen PA, Siren JE, Kuitunen AH, Lindström OK, Kemppainen EAJ (2006). Treatment of abdominal compartment syndrome with subcutaneous anterior abdominal fasciotomy in severe acute pancreatitis. World J Surg.

[44] Leppäniemi A, Mentula P, Hienonen P, Kemppainen E (2008). Transverse laparostomy is feasible and effective in the treatment of abdominal compartment syndrome in severe acute pancreatitis. World J Emerg Surg.

[45] Schachtrupp A, Fackeldey V, Klinge U, Hoer J, Tittel A, Toens C (2002). Temporary closure of the abdominal wall (laparostomy). Hernia.

[46] Fabian TC, Croce MA, Pritchard FE, Minard G, Hickerson WL, Howell RL (1994). Planned ventral hernia. Staged management for acute abdominal wall defects. Ann Surg.

[47] Fansler RF, Taheri P, Cullinane C, Sabates B, Flint LM (1995). Polypropylene mesh closure of the complicated abdominal wound. Am J Surg.

[48] Bleichrodt RP, Simmermacher RK, van der Lei B, Schakenraad JM (1993). Expanded polytetrafluoroethylene patch versus polypropylene mesh for the repair of contaminated defects of the abdominal wall. Surg Gynecol Obstet.

[49] Nagy KK, Fildes JJ, Mahr C, Roberts RR, Krosner SM, Joseph KT (1996). Experience with three prosthetic materials in temporary abdominal wall closure. Am Surg.

[50] Ghimenton F, Thomson SR, Muckart DJ, Burrows R (2000). Abdominal content containment: practicalities and outcome. Br J Surg.

[51] Olejnik J, Vokurka J, Vician M (2008). Acute necrotizing pancreatitis: intra-abdominal vacuum sealing after necrosectomy. Hepatogastroenterology.

[52] Nordback I, Pessi T, Auvinen O, Autio V (1985). Determination of necrosis in necrotizing pancreatitis. Br J Surg.

[53] Lee Y-H, Pratley RE (2005). The evolving role of inflammation in obesity and the metabolic syndrome. Curr Diab Rep.

[54] Porter KA, Banks PA (1991). Obesity as a predictor of severity in acute pancreatitis. Int J Pancreatol.

[55] Karimgani I, Porter KA, Langevin RE, Banks PA (1992). Prognostic factors in sterile pancreatic necrosis. Gastroenterology.

[56] Abu Hilal M, Armstrong T (2008). The impact of obesity on the course and outcome of acute pancreatitis. Obes Surg.

[57] Beger HG, Bittner R, Block S, Büchler M (1986). Bacterial contamination of pancreatic necrosis. A prospective clinical study. Gastroenterology.

[58] De Bernardinis M, Violi V, Roncoroni L, Boselli AS, Giunta A, Peracchia A (1999). Discriminant power and information content of Ranson’s prognostic signs in acute pancreatitis: a meta-analytic study. Crit Care Med.

[59] Talamini G, Bassi C, Falconi M, Sartori N, Frulloni L, Di Francesco V (1996). Risk of death from acute pancreatitis. Role of early, simple “routine” data. Int J Pancreatol.

[60] Lankisch PG, Schirren CA (1990). Increased body weight as a prognostic parameter for complications in the course of acute pancreatitis. Pancreas.

[61] Mery CM, Rubio V, Duarte-Rojo A, Suazo-Barahona J, Peláez-Luna M, Milke P (2002). Android fat distribution as predictor of severity in acute pancreatitis. Pancreatology.

[62] Fain JN, Madan AK, Hiler ML, Cheema P, Bahouth SW (2004). Comparison of the release of adipokines by adipose tissue, adipose tissue matrix, and adipocytes from visceral and subcutaneous abdominal adipose tissues of obese humans. Endocrinology.

[63] Connor S, Raraty MGT, Howes N, Evans J, Ghaneh P, Sutton R (2005). Surgery in the treatment of acute pancreatitis–minimal access pancreatic necrosectomy. Scand J Surg.

[64] Heinrich S, Schäfer M, Rousson V, Clavien P-A (2006). Evidence-based treatment of acute pancreatitis: a look at established paradigms. Ann Surg.

[65] Corfield AP, Cooper MJ, Williamson RC, Mayer AD, McMahon MJ, Dickson AP (1985). Prediction of severity in acute pancreatitis: prospective comparison of three prognostic indices. Lancet.

[66] Chatzicostas C, Roussomoustakaki M, Vlachonikolis IG, Notas G, Mouzas I, Samonakis D (2002). Comparison of Ranson, APACHE II and APACHE III scoring systems in acute pancreatitis. Pancreas.

[67] Ueda T, Takeyama Y, Yasuda T, Matsumura N, Sawa H, Nakajima T (2007). Simple scoring system for the prediction of the prognosis of severe acute pancreatitis. Surgery.

[68] Gardner TB, Vege SS, Chari ST, Pearson RK, Clain JE, Topazian MD (2008). The effect of age on hospital outcomes in severe acute pancreatitis. Pancreatology.

[69] Isenmann R, Rau B, Beger HG (1999). Bacterial infection and extent of necrosis are determinants of organ failure in patients with acute necrotizing pancreatitis. Br J Surg.

[70] Banks PA (1994). Acute pancreatitis: medical and surgical management. Am J Gastroenterol.

[71] Isenmann R, Beger HG (1999). Natural history of acute pancreatitis and the role of infection. Baillieres Best Pract Res Clin Gastroenterol.

[72] Johnson CD, Toh SKC, Campbell MJ (2004). Combination of APACHE-II score and an obesity score (APACHE-O) for the prediction of severe acute pancreatitis. Pancreatology.

[73] Frey CF, Zhou H, Harvey DJ, White RH (2006). The incidence and case-fatality rates of acute biliary, alcoholic, and idiopathic pancreatitis in California, 1994–2001. Pancreas.

[74] Tao H-Q, Zhang J-X, Zou S-C (2004). Clinical characteristics and management of patients with early acute severe pancreatitis: experience from a medical center in China. World J Gastroenterol.

[75] Al-Bahrani AZ, Abid GH, Holt A, McCloy RF, Benson J, Eddleston J (2008). Clinical relevance of intra-abdominal hypertension in patients with severe acute pancreatitis. Pancreas.

[76] De Waele JJ, Hoste E, Blot SI, Decruyenaere J, Colardyn F (2005). Intra-abdominal hypertension in patients with severe acute pancreatitis. Crit Care.

[77] Mentula P, Hienonen P, Kemppainen E, Puolakkainen P, Leppäniemi A (2010). Surgical decompression for abdominal compartment syndrome in severe acute pancreatitis. Arch Surg.

[78] Gecelter G, Fahoum B, Gardezi S, Schein M (2002). Abdominal compartment syndrome in severe acute pancreatitis: an indication for a decompressing laparotomy?. Dig Surg.

[79] Keskinen P, Leppäniemi A, Pettila V, Piilonen A, Kemppainen E, Hynninen M (2007). Intra-abdominal pressure in severe acute pancreatitis. World J Emerg Surg.

[80] Goverman J, Yelon JA, Platz JJ, Singson RC, Turcinovic M (2006). The “Fistula VAC”: a technique for management of enterocutaneous fistulae arising within the open abdomen: report of 5 cases. J Trauma.

[81] Hultman CS, Pratt B, Cairns BA, McPhail L, Rutherford EJ, Rich PB (2005). Multidisciplinary approach to abdominal wall reconstruction after decompressive laparotomy for abdominal compartment syndrome. Ann Plast Surg.

[82] Malbrain MLNG, Chiumello D, Pelosi P, Wilmer A, Brienza N, Malcangi V (2004). Prevalence of intra-abdominal hypertension in critically ill patients: a multicentre epidemiological study. Intensive Care Med.

